# Ergonomic and individual risk factors for musculoskeletal pain in the ageing workforce

**DOI:** 10.1186/s12889-022-14386-0

**Published:** 2022-10-28

**Authors:** Niels-Peter Brøchner Nygaard, Gert Frank Thomsen, Jesper Rasmussen, Lars Rauff Skadhauge, Bibi Gram

**Affiliations:** 1 Research Unit of Health Science, Hospital of South West Jutland, University Hospital of Southern Denmark, Esbjerg, Denmark; 2 Department of Regional Health Research, University of Southern Denmark, Odense, Denmark; 3 Department of Occupational Medicine, Hospital South West Jutland, University Hospital of Southern Denmark, Esbjerg, Denmark; 4 Department of Occupational and Environmental Medicine, Odense University Hospital, Odense, Denmark; 5 Department of Clinical Research, University of Southern Denmark, Odense, Denmark

**Keywords:** Ergonomic exposure, Musculoskeletal pain, Ageing, Work-related posture

## Abstract

**Background:**

The present study aimed to investigate the possible association between specific ergonomic and individual risk factors and musculoskeletal pain (MSP) in the back, shoulder, hip and knee region in workers aged 50-65y.

**Methods:**

The study was a population based cross-sectional survey. The study population comprised citizens born between 1952–1966, living in Esbjerg municipality, Denmark, ultimo 2016 (*n* = 23,463). A questionnaire was sent electronically or by mail. The analysis included the working population only. A multivariate logistic regression was used for each of the following dependent variables; musculoskeletal pain for the past 3 months in the back, shoulder, hip and knee, where independent variables included ergonomic exposure, age, sex, body mass index (BMI) and leisure time physical activity (LTPA).

**Results:**

The overall response rate was 58% and the data of individuals at work (*n* = 9,263) demonstrated several ergonomic exposures with increased odds for pain in specific regions. Exposure to back twisted or bend, squatting or lying on knees and to carrying or lifting were associated with musculoskeletal pain in the back, whereas exposure to back twisted or bend, arms above shoulder and repeated arm movement were associated with pain in the shoulder. Exposure to back twisted or bend, repeated arm movement, squatting or lying on knees and to carrying or lifting were associated with musculoskeletal pain in the hip. Important individual risk factors were also identified. Increasing age was significantly associated with increased pain in the hip but associated with less risk for pain in the back and shoulder. Males had higher odds for pain in the back and knee compared to females but lower odds for pain in the hip. BMI was particularly important for knee pain. The level of LTPA did not have an important association with MSP in any region.

**Conclusion:**

There is a significant positive association between ergonomic exposures and musculoskeletal pain, which were specific for the back, shoulder, hip and knee. In addition, the data demonstrated a differential association with age, sex and BMI. This needs to be considered for the treatment and classification of musculoskeletal pain and for future preventive initiatives.

## Background

The proportion of the workforce above 55y, has increased dramatically in recent decades [1]. Age, irrespective of other factors, has been shown to affect individuals’ ability to work. As individuals age physical and mental health deteriorate [2] causing an imbalance between occupational demands and individuals’ work capacity. This imbalance might have severe consequences with increased risks for disability [3], occupational injury [4], musculoskeletal disorder [5] and poor workability [6] which have important socioeconomic implications. Musculoskeletal pain (MSP) in particular is a prevalent issue [7] and has been shown to cause more absence from work and disability compared to any other group of disease [8]. Importantly, MSPs have been related to both age and work-related ergonomic exposure [5, 9] and occur more frequently in certain occupations such as health care workers [10], manufacturing and industrial work [11], and in construction [12], i.e. occupations involving manual tasks. In addition, MSP has been shown to be a significant risk factor for maintaining health in older age groups [13] and has been associated with, falls, frailty, depression, amongst others [14]. MSP and comorbidities might further interact negatively, exacerbating the impact on work ability, quality of life and mortality [15]. MSP is common, underreported and often inadequately treated in the older age groups leading to mismanagement and chronicity [14]. It is thus imperative to further delineate the complex interaction between ergonomic exposure at the workplace and MSP in the oldest group of workers.

The deleterious effects of being exposed to high ergonomic load is well-known, however, the difference in effects of being physically active at work vs. leisure time, is a paradox [16]. Physical activity is generally considered to be beneficial by maintaining physical capacity, reducing MSP and preventing lifestyle related disease. However, it is becoming increasingly clear that work related physical activity can indeed impair health [5]. For example, manual work in awkward positions, with many repetitions and heavy lifting have been linked to pain in the shoulder, back and hip / knee [8] and a recent systematic review suggests that the occupational exposure to some of these risk factors remains highly prevalent [17]. Ageing is associated with an attenuation of physical capacity and mental health [2]. In this line, depending on individuals’ lifestyle, body weight and genetics [18], there is a substantial decrease in muscle strength [19], bone density and aerobic capacity, resulting in a steep decline in functional capacity especially at the age of 60 and above [20]. These physiological and mental changes might have an important impact on the balance between job requirements and individual job capacity, especially when the physical demands are high [9].

Regarding pain, multiple occupational and non-occupational risk factors, such as leisure time physical activity (LTPA) [21], systemic disease, obesity or stress might be relevant. Thus, the etiology is multifactorial with interacting biological, psychological and social factors [22] and it is key to clarify the factors that might account for MSP, in what region and to what extent. So far, results vary. Exposures is often dichotomized, hampering the interpretation of the exposure–response relationships. There are also differences in methodology, and differences in the definition of exposures and data available for analysis. Studies on MSP often focus on long term sickness absence [23] which is indeed crucial but also lacks the degree of specificity needed for targeted preventive initiatives and treatment in occupational medicine. This is further highlighted by the lack of effective interventions at the workplace [24]. In many cases, one of the underlying causes for long term sickness absence might be MSP in a specific region, and more efforts should be done to elucidate the dynamic and intensive interaction between personal resources, ergonomic exposures and MSP, particularly in the oldest group of workers. A better understanding of these issues is crucial to focus preventive measures aiming to ensure workers’ wellbeing, as well as their continued attachment to the labor market.

The present study aimed to investigate the possible association between specific ergonomic and individual risk factors for workers aged 50-65y and MSP in the back, shoulder, hip and knee region. The study was part of a previous study (The Esbjerg Cohort), previously described [6]. We hypothesized that ergonomic exposure, independently of other variables, would be associated with MSP and that these exposures would be region specific. We further hypothesized region specific associations with personal factors including age, sex, LTPA and BMI.

## Methods

### Study design

This present study is part of a population based cross-sectional survey conducted in the 4^th^ quarter of 2017 – 2^nd^ quarter of 2019 in Esbjerg municipality [6]. The methodology has been described elsewhere [6]. In brief, a comprehensive questionnaire was constructed, based on validated questionnaires, focusing on health status, musculoskeletal pain, perceived stress, ergonomic exposure and workability. The present study investigates the association between ergonomic exposure and MSP in the oldest group of workers and all methods were performed in accordance with the relevant guidelines and regulations.

### Ethics

The study was registered with The Danish Data Protection Agency (file no. 2008–58-0035). The need for formal ethical approval was waived by The Regional Committees on Health Research Ethics for Southern Denmark (file nr: S-20180162) because the study did not involve biomedical interventions. Finally, members from a panel of patients and relatives, discussed and approved the content and setup of the study. Data were anonymized and analyzed based on code identifiers.

### Participants

Names and social security numbers of citizens born between 1952 and 1966 living in the Esbjerg municipality in December 2016 (*n* = 23,463) were obtained from the Danish Health Data Authority. A questionnaire was sent electronically, when possible, to their public electronic mailbox (Eboks), otherwise by conventional mail. The questionnaire was sent again in case of no response, resulting in a response from 13,599 individuals (response rate ~ 58%). Data were collected using the REDCap electronic data capture tool (OPEN, University of Southern Denmark) [25]. The present study included individuals that reported to be employed or self-employed when answering the questionnaire.

### Outcome variable

#### Musculoskeletal pain

The present study focused on MSP in the body regions: back, shoulder, hip and knee. The Standardized Nordic Questionnaire (SNQ) [26] was used to obtain the average pain score for the past 3 months, as measures by a visual analogue scale (VAS), where 0 was defined as “no discomfort” and 100 was defined as worst possible pain and discomfort for each region. The scores were dichotomized into no pain (VAS 0–39) and pain (VAS 40–100) [27].

### Predictor variables

#### Ergonomic exposure

Estimation of physical work demands were assessed with eight questions: During the working day – to which extent do you: a) sit, b) walk or stand, c) work with your back bent / twisted without hand- and arm support, d) have your arms raised to or above shoulder height, e) perform repetitive arm movements several times per minute (e.g. package work, mounting, machine feeding, carving), f) squat or kneel when you work, g) push or pull, h) carry or lift. The answer categories were: 1) almost all the time, 2) approximately ¾ of the time, 3) approximately ½ of the time, 4) approximately ¼ of the time, 5) rarely/very little, or 6) never. The questions were further categorized into low (5 + 6), moderate (3 + 4) and high exposure (1 + 2) respectively. Question a was left out of the analysis since it was an antagonist to question b.

#### Individual risk factors

Respondents were divided in gender and categorized in three age groups: 50–55, 56–60, and > 60 years. BMI was calculated using the respondents’ weight in kilograms divided by the square of height in meters (kg/m^2^), and categorized into underweight (< 18,5), normal (18.5–24.9), overweight (25.0–29.9), obese (30.0–34.9) and extremely obese (> 40.0). To evaluate LTPA, participants were asked to describe their level of leisure physical activity on the basis of two categories: a) recreational sports, heavy gardening, or fast walking / cycling where you sweat or get short of breath, b) high intensity training or competitive sports, according to the following response options: 1) does not perform the activity, 2) under 2 h per week, 3) 2–4 h per week and 4) more than 4 h per week.

### Control variables

Work-related stress was assessed using the Danish version of the 10-item Perceived Stress Scale (PSS-10) [28]. PSS-10 scores were obtained by reversing the scores on the four positive items, e.g., 0 = 4, 1 = 3, 2 = 2, etc. and then summing across all 10 items. Items 4, 5, 7, and 8 were the positively stated items. The summarized score was categorized into low (0–13), moderate (14–26) and high (27–40) stress. Chronic disease included cardiovascular disease, cancer, diabetes, depression, asthma, chronic obstructive pulmonary disease, metabolic disease. These diseases were assessed with the categorical options “Yes” and “No” and respondents were categorized as having chronic disease, having answered “Yes” to any of the above. Finally, smoking status was assessed with the question: “Do you smoke tobacco” with the following categorical variables “Yes”, “No”, and “Previously”.

### Statistical analyses

The analyses and statistics were performed using the statistical software Stata16 (StataCorp, USA). Demographics of the population are presented as prevalence and percentage. Multivariate logistic regression was used to estimate the associations between MSP (dependent variable) and ergonomic – and individual risk factors (independent variables). Multivariate logistic regression was performed for each region, i.e., the back, shoulder, hip and knee, and included all predictor and control variables described above. Results are reported as Odds Ratio (OR) and 95% confidence intervals (CI) unless otherwise stated, using a forest plot. Variables with CI’s not overlapping 1 was considered statistically significant. The model did not impute missing values.

## Results

**Fig. 1:**
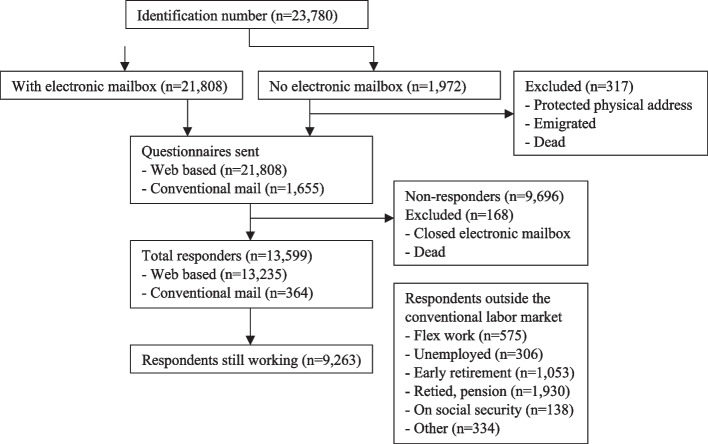
Flow diagram. Depicts the number of individuals identified in the Esbjerg municipality and the number of respondents to the questionnaire

In December 2016, a total of 23,780 citizens with year of birth between 1952–1966 were identified in the Municipality of Esbjerg, Denmark. Among those, 21,808 had a valid Eboks and received a web-based questionnaire (Fig. 1) and of the remaining 1,972 persons, it was possible to retrieve a valid postal address for 1,655 persons from Statistics Denmark. Eleven persons had emigrated, two had disappeared, one person changed identity, 10 were unknown at the address, 13 had protected address and 280 had passed away before retrieval of the postal addresses leaving a total of 23,463 persons eligible for the study. After one reminder, 13,599 (58%) individuals had answered the questionnaire of which a total of 9,263 (68%) stated to be at work when answering the questionnaire. In Esbjerg Municipality 65% of the population aged 50–64 were at work [43], showing a very modest over representation of being at work among the responders. The demographics and reported health of the population are presented in Table 1.

**Table 1:** Descriptive statistics of the study population—citizens between 50-65y living in the Esbjerg municipality in December 2016

*Parameters*	**Frequency (n)**	**Percentage (%)**
Sex		
Male	4681	50.5
Female	4582	49.5
Age group		
50-55y	3253	35.1
56-60y	3931	42.4
> 60y	2079	22.4
Work type		
White collar	6929	74.8
Blue collar	2334	25.2
MSP		
Back pain	2102	22.7
Shoulder pain	1745	18.8
Hip pain	758	8.2
Knee pain	1204	13.0
Walk / stand		
Low exposure	999	10.8
Moderate exposure	4038	43.6
High exposure	3667	39.6
Back twisted / bend		
Low exposure	5504	59.4
Moderate exposure	2259	24.4
High exposure	977	10.6
Arms above shoulder		
Low exposure	6802	73.4
Moderate exposure	1659	17.9
High exposure	293	3.2
Repeated arm movement		
Low exposure	6177	66.7
Moderate exposure	1466	15.8
High exposure	1089	11.8
Squatting / lying on knees		
Low exposure	7137	77.1
Moderate exposure	1424	15.4
High exposure	204	2.2
Pushing /pulling		
Low exposure	6504	70.2
Moderate exposure	1822	19.7
High exposure	405	4.4
Carrying / lifting		
Low exposure	5860	63.3
Moderate exposure	2329	25.1
High exposure	571	6.2
Moderate LTPA		
None	1602	17.3
Under 2 h/w	3400	36.7
2–4 h/w	2344	25.3
> 4 h/w	1080	11.7
Intense LTPA		
None	6880	74.3
Under 2 h/w	715	7.7
2–4 h/w	416	4.5
> 4 h/w	225	2.4
BMI		
Underweight	48	0.5
Normal	3095	33.4
Overweight	3484	37.6
Obese	1673	18.1
Extremely obese	142	1.5
Smoking		
Yes	1457	15.7
Previously	1747	18.9
Never	5330	57.5
Chronic cardiovascular disease		
Yes	350	3.8
Diabetes		
Ye	452	4.9
Asthma		
Yes	776	8.4
Metabolic disease		
Yes	500	5.4
Depression		
Yes	216	2.3
Cancer		
Yes	701	7.6
COPD		
Yes	285	3.1

*Abbreviations*: *MSP* Musculoskeletal pain, *LTPA* Leisure time physical activity, *BMI* Body mass index, *COPD* Chronic obstructive pulmonary disorder

Low exposure indicates 0–25% of the time, moderate exposure = 25–50% of the time, high exposure = 75% or more of the time. MSP was dichotomized into no pain (VAS 0–39) and pain (VAS 40–100)

### Ergonomic risk factors

**Fig. 2:**
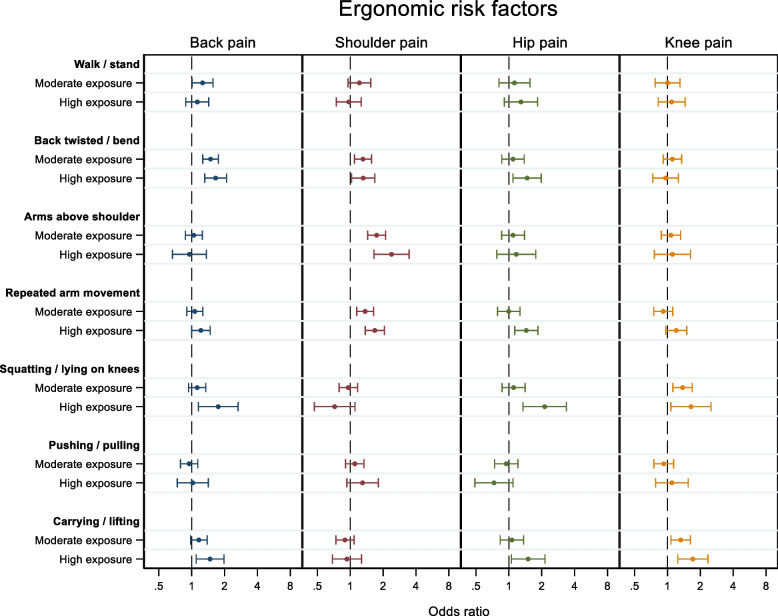
Shows a forest plot of the OR and 95% CI for ergonomic stressors (independent variables) for each painful region (dependent variables) back (blue), shoulder (red), hip (green) and knee (yellow), adjusted for age, BMI, LTPA, stress, chronic disease and smoking. The OR indicates the odds for having a VAS pain score = for each region, adjusted for all other variables. Statistically significant differences (*p* < 0.05) from reference level are apparent when 95% CI does not overlap the dotted line (x = 1). For clarity, reference levels were left out of the figure for the independent variables

There was a significant association between a number of ergonomic risk factors and MSP dependent on the anatomical region (Fig. 2).

Work-related walking and standing 25–50% of the time (moderate exposure), compared to 0–25% of the time (low exposure), increased the odds for having a pain intensity score = 40 in the back [OR 1.26, 95% CI 1.01–1.57]. There were no significant association for shoulder, hip, or knee pain.

Working with the back twisted / bend had a significant association with pain in both the back, shoulder and hip. The most pronounced effects were observed for the back, showing increased odds for back pain when working 25–50% of the time and 75% of the time (high exposure) or more with the back twisted or bend [OR 1.49, 95% CI 1.26–1.76 and OR 1.66, 95% CI 1.32–2.09, respectively]. For the shoulder, the data similarly showed significantly increased odds for pain working 25–50% of the time and working 75% of the time or more with the back twisted or bend [OR 1.31, 95% CI 1.09–1.56 and OR 1.31, 95% CI 1.03–1.68]. Finally, the odds for having hip pain also significantly increased when exposed to work with the back twisted or bend but only when exposed for more than 75% of the time working. There was no association with knee pain when exposed to the back twisted or bend.

When exposed to work with arms above shoulder height, the results showed significantly higher odds for shoulder pain, both when exposed 25–50% of the time [OR 1.74, 95% CI 1.44–2.11] and 75% or more of the time [OR 2.4, 95% CI 1.65–3.46]. There were no association with neither back, hip nor knee pain when exposed to work with arms above shoulder height.

Similarly, repeated arm movement similarly showed significantly higher odds for shoulder pain, when exposed 25–50% of the time [OR 1.37, 95% CI 1.14–1.64] and 75% or more of the time [OR 1.68, 95% CI 1.37–2.05]. In addition, there were significantly higher odds for hip pain when exposed to repeated arm movement 75% or more of the time [OR 1.44, 95% CI 1.13–1.84]. There were no association with back or knee pain when exposed to repeated arm movement.

When exposed to squatting or lying on knees, the odds for having knee pain increased significantly both when exposed for 25–50% of time [OR 1.37, 95% CI 1.12–1.68] and for 75% or more [OR 1.64, 95% CI 1.08–2.50]. When squatting or lying on knees for 75% of time or more, the odds for pain also significantly increased for the back [OR 1.75, 95% CI 1.15–2.66] and hip [OR 2.13, 95% CI 1.35–3.36].

Carrying or lifting for 25–50% of the time and for 75% or more showed significantly increased odds for knee pain [OR 1.32, 95% CI 1.08–1.62 and OR 1.71, 95% CI 1.24–2.35, respectively]. Exposure for 75% of the time or more showed significantly increased odds for pain in the back [OR 1.47, 95% CI 1.10–1.98] and hip [OR 1.50. 95% CI 1.05–2.14]. There were no association with shoulder pain.

Exposure to pushing or pulling did not change the odds for pain in any region.

### Individual risk factors

**Fig. 3:**
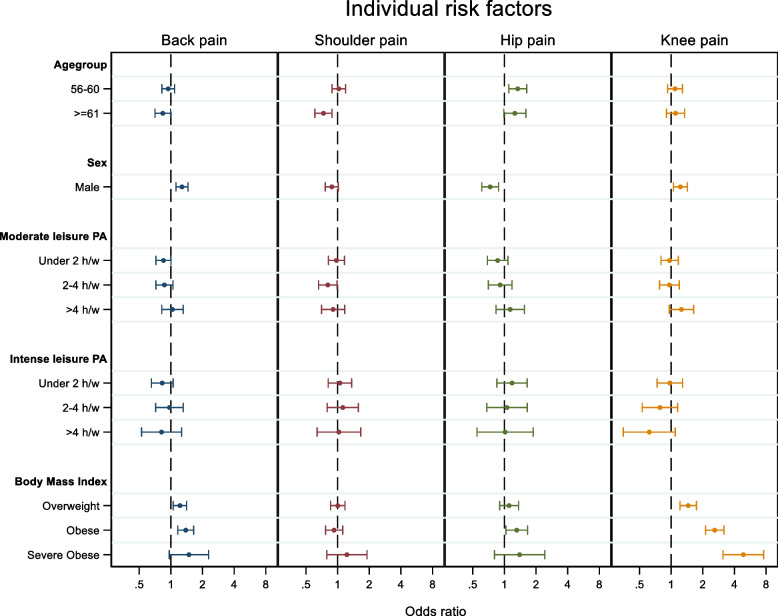
Shows a forest plot of the OR and 95% CI for personal stressors (independent variables) for each painful region (dependent variables) back (blue), shoulder (red), knee (green) and hip (yellow), adjusted for ergonomic exposures, stress, chronic disease and smoking. The OR indicates the odds for having a VAS pain score = 40 for each region, adjusted for all other variables. Statistically significant differences (*p* < 0.05) from reference level are apparent when 95% CI does not overlap the dotted line (x = 1). For clarity, reference levels were left out of the figure as well as the underweight category for BMI

Similar to ergonomic exposures, a number of individual risk factors showed a significant association with pain dependent on the region (Fig. 3).

For age, being > 60y, the odds for back pain [OR 0.84, 95% CI 0.71–0.99] and shoulder pain [OR 0.73, 95% CI 0.61–0.88] significantly decreased compared to being 50-55y. In contrast, being 56-60y significantly increased the odds for hip pain [OR 1.34, 95% CI 1.10–1.63] compared to being 50-55y.

Males showed significantly increased odds for back pain [OR 1.28, 95% CI 1.12–1.46] and knee pain [OR 1.23, 95% CI 1.05–1.43] compared to females. In contrast, males showed significantly decreased odds for hip pain compared to females [OR 0.73, 95% CI 0.61–0.88].

Limited effects were observed in terms of LTPA. Moderate intensity LTPA for 2–4 h/w showed significantly decreased odds for shoulder pain [OR 0.81, 95% CI 0.66–0.99]. No other associations were observed for neither moderate nor intense LTPA.

BMI had a significant association with back, hip, and knee pain. Looking at back pain, being overweight [OR 1.22, 95% CI 1.05–1.41] and obese [OR 1.38, 95% CI 1.16–1.65] showed significantly higher odds for pain. For the hip, only obese showed increased odds for pain [OR 1.31, 95% CI 1.03–1.66]. Finally, knee pain was particularly associated with BMI, showing significantly increased odds for pain being overweight [OR 1.45, 95% CI 1.21–1.74], obese [OR 2.60, 95% CI 2.13–3.17] and severely obese [OR 4.86, 95% CI 3.11–7.59] compared to normal weight. There were no association between BMI and shoulder pain.

Stress, smoking, depression and chronic disease were primarily used to control for confounding effects. Stress was associated with pain in all regions. Smoking was associated with back pain but not with any of the other regions. Depression was not associated with pain in any region. Chronic disease was associated with increased odds for pain in the back and knee but not for the shoulder or hip.

## Discussion

The aim of the present study was to investigate the association between ergonomic exposure and MSP in the back, shoulder, hip and knee for the oldest group of workers aged 50-65y. The study identified ergonomic exposures with increased odds for pain in specific regions. Important individual factors were also identified and were also region specific. Males had higher odds for pain in the back and knee compared to females whereas they had lower odds for pain in the hip. BMI was particularly important for knee pain and LTPA did not have an important association with MSP in any region. Importantly, associations were region specific allowing for further clarification of etiology, prevention and treatment.

The present study includes a large sample representative of the general working population, which strengthens the statistical power considerably. However, it should be acknowledged that the present study has some limitations. The study focuses on the population still at work and thus might exclude vulnerable individuals already outside of the labor market. This may cause a significant bias in the results, known as the “healthy worker effect”. It is also important to note that while the cross-sectional design allows for multiple outcomes to be studied, it does not allow for an interpretation of any causal effects. The results show associations between a large set of parameters in a large population which can be used for further hypothesis generation and perhaps, with caution, some general directional guidelines. Similarly, self-reported data includes a certain amount of variability and uncertainty due to validity issues, recall bias, and a priori knowledge of disease status which might lead to reporting bias.

### Ergonomic risk factors

In summary, the ergonomic exposures associated with a) *back pain* included walking and standing 25–50% of the time, exposure to back twisted or bend for more than 25% of the time, squatting or lying on knees for more than 75% of the time and to carrying or lifting for more than 75% of the time b) *shoulder pain* included exposure to back twisted or bend, arms above shoulder and repeated arm movement for more than 25% of the time, c) *knee pain* included squatting or lying on knees and to carrying or lifting for more than 25%, d) *hip pain* included exposure to back twisted or bend, repeated arm movement, squatting or lying or knees and carrying or lifting for more than 75% of the time.

Moderate exposure to walking or standing, between 25–50% of the work time, was in the present study only associated with back pain. Standing has been reported to reduce blood supply to the muscles, accelerating fatigue and discomfort, thus changing the activity of the muscles and the postural stability [29]. This have been shown to impose health risks such as cardiovascular problems, musculoskeletal disease and long-term sick leave [30]. The significant association with pain in the back region was in line with Sterud et al. 2013, who in a prospective study of the general working population, reported prolonged standing as an important predictor for low back pain [31]. Nevertheless these results remain conflicting [32] and the present study did not observe any statistically significant associations with walking for more than 75% of the time and MSP. Other authors have shown a significant association with other regions, such as the hip or knee [33] and this discrepancy between studies is likely explained by methodological differences and the complex relationship between standing, walking and sitting. Including standing and walking in the same category might further confound the results, since these in part counteract each other.

Working with the back twisted or bend more than 25% of the workday was associated with pain in multiple regions, i.e., the back (moderate and high exposure), shoulder (moderate and high exposure) and hip (high exposure). Working with the back twisted or bend, includes one third of the participant in the present study and is a common exposure apparent in many different occupations and might have important implications for future interventions. It has also been linked to increased risk for long term sickness absence which makes sense since this exposure increases the risk for significant pain in multiple anatomic regions as shown in the present study and by other authors [23]. Working with the back twisted or bend has been associated with increased intradiscal pressure increasing the risk for degeneration or hearniation of the spinal discs [34] and has been classified as a hazardous activity [35], particularly when there is an imbalance between physical capacity and exposure to ergonomic stressors [36]. This imbalance explains, in part, the significant association with pain in the back and ergonomic exposure, that was observed in the present study and in other previous studies [37]. The present study also found a significant association with working with the back twisted or bend and hip pain. This relationship was less clear in present study, although pain in the hip has been associated with physically demanding work in general [38]. The present study demonstrated an association of working with the back twisted or bend with pain in the shoulder. Previous studies have showed that working in awkward postures, is associated with pain in the shoulder [39]. Mechanisms include muscle fatigue [40], prolonged muscle activation [41], inflammatory processes [42], reduced microcirculation [41], static and repetitive mechanical pressure on tendons [43]. Shoulder pain is widespread and has high persistence rates [44]. In this line the present study similarly showed a significant association with working with the arms above shoulder height and with repeated arm movement. Working with arms above shoulder levels has been shown to be an important predictor for shoulder pain previously [44], nevertheless results are not consistent across studies [44]. Similarly, repeated arm movement has been shown to be associated with pain in the shoulder [45], and it has been suggested that the shoulder is prone to injury due to its complex structural architecture, especially when exposed to excessive load and repetitive activity that might precipitate tear, degeneration and tendinopathy, compromising stability and function [46]. This also affects etiology and pathogenesis, which remains controversial and is likely multifactorial.

Expectedly, squatting / lying on knees was particularly associated with pain in the knee showing increased odds at both moderate and high exposure levels which was in line with others [47]. During such exposure the forces around the knee are high, inducing persistent strain on the anatomical structures [48]. This includes increased varus moments that has been associated with misalignment and pain [49] and cumulative mechanical strain [47]. Over time, pain might arise due to inflammatory and degenerative arthritis, bursitis and injury to cartilage ligaments and other surrounding structures.

Interestingly, squatting / lying on knees was also associated with pain in the back and hip. Back pain has previously been associated with squatting and kneeling [30], as is the case with pain in the hip [38]. Generally, asymmetric activity around the hip joint might cause non-optimal adaptations, causing sacroiliac dysfunction and is closely related to pain in the back [50].

There were no statistically significant results for pushing / pulling, which was surprising. Previous studies have associated pushing / pulling with both pain in the back and shoulder [51] and also for the knee [52]. In this regard, it should be noted that the present study included all ergonomic exposures in the statistical model, and because these have a relatively high correlation, there is an increased risk for overadjustment bias. This necessitates careful interpretation of the results and might explain some of the discrepancies observed for pushing / pulling and other ergonomic exposures.

In contrast, carrying / lifting was associated with back, hip and knee pain. Lifting has been associated with high mechanical loads, moments and spinal compression forces [53] and previous studies also confirm the results in the present study showing similar association with both back pain [54], hip pain [55] and knee pain [56]. There were no association with shoulder pain which was in contrast to others [30]. The differential effect between pushing / pulling and carrying / lifting might underline the marked difference between the two from a biomechanical point of view. However self-report might have resulted in misclassification of the exposures causing biased results. Objective measurement methods might be needed to obtain a sufficient level of detail, as in for example Hoozemans et al. 2002 [51]. In addition, the present study employed a mutually adjusted regression model that included all ergonomic exposures which require careful interpretation and might further explain the discrepancy between studies.

In general, the above exposures are conceptually vaguely described, and many are dynamic, highly variable and can be quantified by both duration, frequency and intensity, that affect biomechanical load differently. Also, a combination of exposures is likely important. For example Miranda et al. (2008) observed that a combination of force, posture and overhead work increased the risk for clinically diagnosed shoulder disorder fourfold [39]. Finally, psychosocial factors might be important [57] and a lack of worker control of for example work schedule and environment [16]. Taking all these factors and the study design into account, it is clear that the present study cannot infer causality, which remains a major challenge in this area [32]. Nevertheless, the data suggest that exposure to work-related physical activity and strenuous postures at work does not benefit the health of the oldest group of workers. Muscular disorders are highly prevalent [58] with poor general health, reduced work ability [6] and sickness absence [23].

### Individual risk factors

The present study showed significant associations with individual factors such as gender, BMI and age which might explain the high background prevalence of MSP in the population in general. Interestingly, age was not a strong risk factor for MSP. Only pain in the hip was significantly associated with increasing age whereas age was associated with less risk for pain in the back and shoulder. One explanation is the impact of a healthy worker effect. Increasing pain might force workers into new occupations which can make interpretation difficult. Other authors have shown that it is possible to compensate, in part, for pain and ergonomic exposures [59].

The present study demonstrated important and differential associations between sex and MSP. Males had significantly higher odds for pain in the back and knee compared to females which is in contrast to prior research [60]. Higher prevalence for MSP are generally observed in females and has been attributed to psychological factors [61], such as a higher somatization [62]. Also differences in muscle strength and work environments designed primarily for men have also been cited as possible explanations [63]. In this line the present study showed that females had significantly higher odds for pain in the hip. This has been observed previously [38] and has been linked to specific changes causing laxity in spine and pelvic structures [64]. Sex discrepancies have been observed for shoulder pain [39] and the present study observed a similar directional pattern although not statistically significant. These results might further indicate that differential effects occur between sex and ergonomic exposure, however, no interaction effects (sex#ergonomic exposure) were observed, except at high exposure to pushing / pulling (data not shown). In general, additional studies are needed to further elucidate the differential association between sex, ergonomic exposure and MSP. One strategy is to utilize stratified analyses to derive specific changes related to sex depending on ergonomic exposures, which was outside the scope of the present study.

Surprisingly, this study did not show a significant association between LTPA and MSP. The effects of LTPA on health markers in workers with high physical demands at work remain controversial [65]. Research does suggest that LTPA is beneficial for overall health of workers and for their workability [66] but the present study show a less clear association with respect to MSP. According to Norheim and colleagues, individuals that performed LTPA, had lower odds for low back pain and pain in the hips and knees which is in contrast to the present study, while others demonstrate results that are in line with the present study [67]. This discrepancy is likely explained by differences in methodology, formulation and construction of questions and also by the inherent variability and lack of specificity for patient reported outcomes of physical activity [68]. Further, some sports are negatively associated with musculoskeletal pain which was not evaluated in the present study [69].

The present study showed that BMI was important for pain in the back, hip and particularly the knee which confirms previous findings [70]. This link can be explained by increased mechanical demands [71], particularly for the weight-bearing joints, as shown in the present study, by metabolic changes [72] and by impaired ventilatory function [73] which was not part of the present study. Nevertheless, the association is complex, and discrepancies exist. For example, studies have shown no association with back pain [70] and others have found significant association for the upper body [70], which is in contrast to the present study.

### Perspectives / Practical implications

Long-term exposure to work with high physical demands might increase the age dependent deterioration of physical capacity, which may in turn affect workers ability to cope with specific ergonomic exposures. This has important implications for future guidelines and regulation. To ensure safety, quality of life, good health and the continued participation of the oldest group of workers in the labor market, a better understanding of age-related changes and its interaction with the cumulative exposure to risks such as high physical demands is required. The determinants of health and work ability are multifactorial and relates to both physical and psychosocial factors within and outside the workplace, which makes workplace interventions complex to design and implement but also interpret. This study provides some of the pieces necessary for properly targeted preventative initiatives for workers at risk and contributes to a clarification of the etiology of work-related disease and in the classification, treatment, and prognosis of patients. This includes preventive interventions specifically designed and targeted for individual anatomic regions and special attention on individual factors such as sex and BMI.

## Conclusion

The present study showed that both ergonomic work exposure and individual factors have an important effect on the risk for developing MSP and that it is region specific. Ergonomic exposures such as back twisted / bend, carrying / lifting and squatting / lying on knees, were associated with pain in multiple regions and might therefore be of particular interest for further research and interventions. The data further suggest that sex needs to be accounted for in clinical settings and when designing workplace interventions and that, aside of ergonomic exposure, BMI might be a target of interest for such interventions.

## Data Availability

The data presented in this study are available on request from the corresponding author (NPBN). The data are not publicly available due to privacy and ethical reasons.

## Abbreviations

MSP: Musculoskeletal pain
BMI: Body mass index
LTPA: Leisure time physical activity
CI: Confidence interval
OR: Odds ratio
VAS: Visual analogue scale
